# Combined treatment with surgery and immune checkpoint inhibitor extended survival in a case of gastric intramural metastasis from esophageal cancer: a case report

**DOI:** 10.1186/s40792-023-01703-x

**Published:** 2023-06-26

**Authors:** Ayako Wakasugi, Yasue Kimura, Keita Natsugoe, Tomonori Nakanoko, Kentaro Nonaka, Sho Nambara, Qingjiang Hu, Ryota Nakanishi, Mitsuhiko Ota, Eiji Oki, Yoshinao Oda, Tomoharu Yoshizumi

**Affiliations:** 1 Department of Surgery and Science, Graduate School of Medical Sciences, Kyushu University, 3-1-1, Maidashi, Higashi-Ku, Fukuoka, 812-8582 Japan; 2 Department of Histopathology, Kyushu University Hospital, Fukuoka, Japan

**Keywords:** Esophageal cancer, Nivolumab, Gastric intramural metastasis

## Abstract

**Background:**

Intramural metastasis (IM) of esophageal cancer is classified as distant metastasis according to the Japanese Classification of Esophageal Cancer, and it is well-known to be associated with a poor prognosis. We herein report a case of perforated gastric IM of esophageal cancer that was successfully controlled with nonradical surgery and subsequent immune checkpoint inhibitor (ICI) treatment.

**Case presentation:**

A 72-year-old woman was referred to our department for the treatment of esophageal cancer and perforated gastric ulcer. A histological examination of the main tumor and gastric ulcer lesion revealed squamous cell carcinoma. Since the gastric wall tumor had invaded the celiac artery, complete resection was considered impossible. Chemotherapy was administered but led to severe adverse events, so palliative resection was performed. Two months after surgery, computed tomography revealed enlargement of the residual tumor around the celiac artery. However, after nivolumab monotherapy was started, the tumor diminished remarkably, and the quality of life of the patient dramatically improved. Nine months after nonradical surgery, she is surviving without any disease concern.

**Conclusions:**

With the increased availability of ICIs, multidisciplinary treatment with surgery and ICIs can potentially lead to long-term survival, even in cases expected to have a poor prognosis.

## Background

Gastric intramural metastasis (IM) of esophageal cancer is associated with a poor prognosis [1, 2] and is considered distant metastasis according to the Japanese Classification of Esophageal Cancer due to its biological grade [3, 4]. We recently encountered a case involving gastric perforation caused by gastric IM from esophageal cancer. The patient was successfully treated using a combination of palliative surgery and postoperative treatment with an immune checkpoint inhibitor (ICI).

## Case presentation

**Fig. 1:**
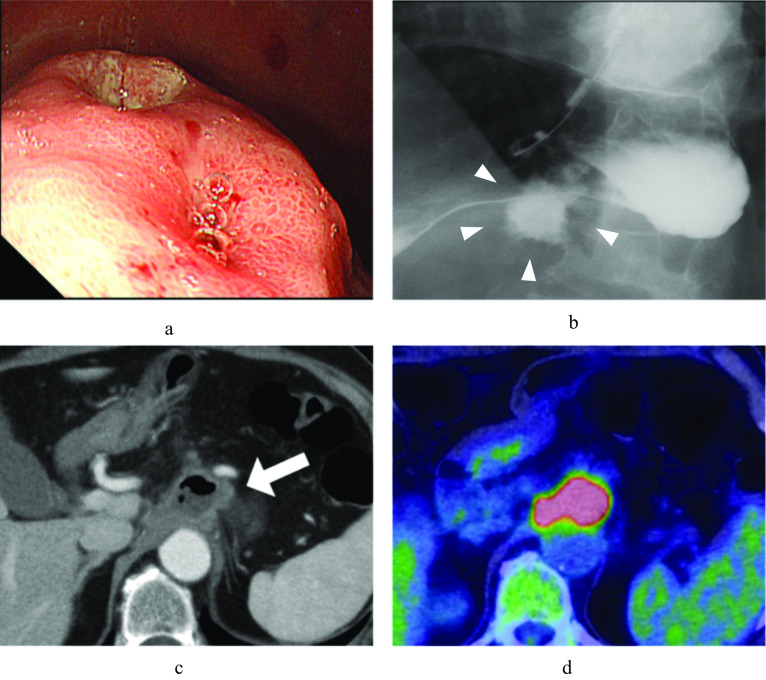
Submucosal tumor with depression was observed at the lesser-posterior side of the gastric wall (**a**). Leakage of contrast medium at the lesser side of the stomach (**b**). Cystic mass at the front of the celiac artery (**c**). FDG accumulation was observed at the cystic mass on positron emission tomography (**d**)

A 72-year-old woman was referred to our department with a diagnosis of thoracic esophageal cancer and perforated gastric ulcer. Imaging studies showed fluid accumulation outside the gastric wall, a cystic mass that was suspected of being an abscess caused by gastric perforation, accumulation of fluorodeoxyglucose (FDG) in the affected area, and no signs of distant metastasis (Fig. 1). A biopsy confirmed squamous cell carcinoma in the esophagus and an ulcerative lesion at the gastric wall.

**Fig. 2:**
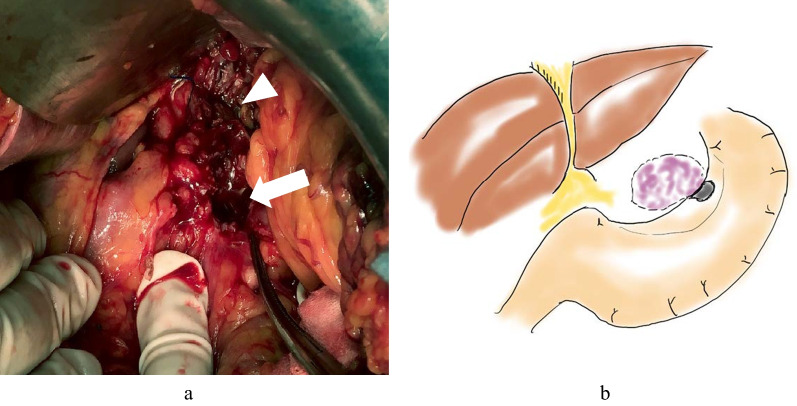
Surgical findings (**a**). Necrotic tissue was found in front of the celiac artery (white triangle). Perforation site at the lesser side of the stomach (white arrowhead) and a schematic illustration with surgical findings are shown (**b**)

To enable oral intake, gastrectomy was needed. However, the feasibility of the surgical approach remains debatable given the high likelihood of residual malignancy. Chemotherapy was administered with a regimen combining docetaxel, cisplatin, and 5-fluorouracil (day 1 and day 15: docetaxel 30 mg/m^2^, day 1: cisplatin 80 mg/m^2^, day 1 to day 5: 5-fluorouracil 800 mg/m^2^). On day 15, docetaxel could not be administered due to neutropenia and a decreased performance status. However, the patient refused the second cycle of chemotherapy, because she developed strong adverse events, including diarrhea and febrile neutropenia. Robot-assisted thoracoscopic esophagectomy and open laparotomy were performed for resection and drainage of the cystic mass, but we were unable to completely resect it as expected due to celiac artery invasion by a mucus-filled cystic mass (Fig. 2). The perforation site was within the area to be resected at the time of gastric tube creation, so we estimated that it would not interfere with gastric tube reconstruction, so we performed esophageal reconstruction with a narrow gastric tube. We considered colon reconstruction and two-stage reconstruction of the small intestine, but considering the progress of the cancer, we chose this operative procedure that would allow early reconstructive oral intake.

**Fig. 3:**
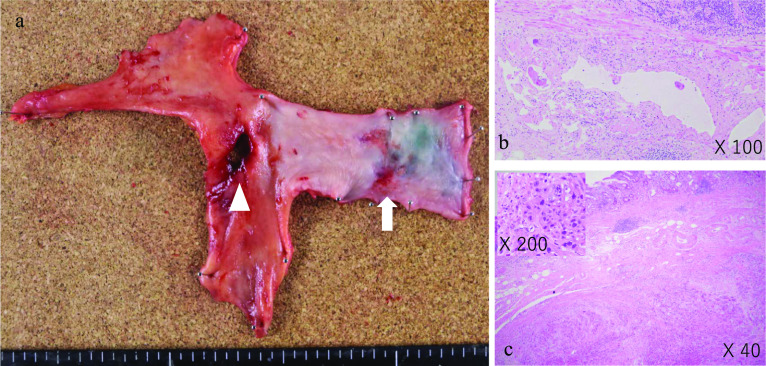
Macroscopic pathological findings of the specimen (**a**). Primary lesion of the esophagus (white arrowhead) and perforation site of the stomach (white triangle). Microscopic histopathological findings show lymphatic vessel invasion in the esophageal primary lesion (**b**). Squamous cell carcinoma is seen at a deeper site of the submucosa within the gastric wall (**c**)

**Fig. 4:**
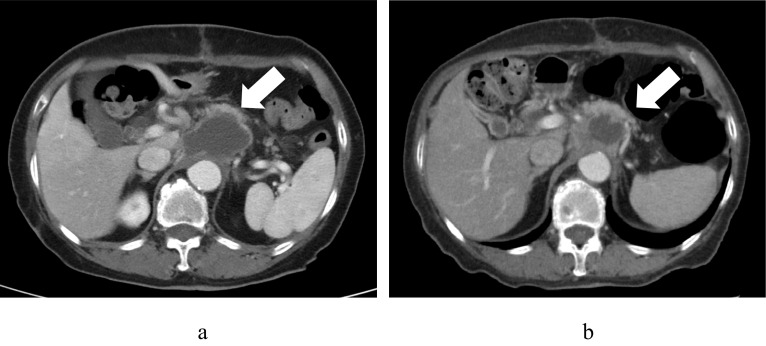
Residual tumor was enlarged (**a**). After three courses of nivolumab, the enlarged residual tumor size was reduced (**b**)

The patient was discharged home without any complications. A postoperative pathological examination showed lymphatic invasion in the primary esophageal lesion and squamous cell carcinoma in the deep submucosal layer of the gastric wall, with no evidence of lymph node involvement in the perforated gastric wall (Fig. 3), and the ulcerative lesion in the stomach was determined to be an IM. Residual tumor enlargement was detected on computed tomography (CT) performed 2 months after surgery. The patient was treated with nivolumab 240 mg/body as second-line therapy, which reduced the tumor size and dramatically improved her quality of life (Fig. 4). Nine months after the nonradical surgery, she is surviving without any disease concern.

## Discussion

IM of esophageal cancer, especially gastric IM, is considered distant metastasis according to the Japanese Classification of Esophageal Cancer due to its high malignancy grade and is a poor prognostic factor [3, 4]. IM from esophageal cancer was first reported in 1933 [5], and its mechanism has been suggested to be metastasis of submucosal cancerous tissue via the lymphatic system [6, 7]. An accurate preoperative diagnosis of gastric IM can be difficult due to its submucosal tumor-like morphology and tendency to be larger than the primary lesion in the gastric fornix and upper part of the body [8].

While esophageal IMs can be diagnosed endoscopically, gastric IM of esophageal cancer is challenging to diagnose. Surgery is often performed in cases where the preoperative diagnosis is made if there are no other metastases, but whether resection or chemotherapy is the optimal treatment approach remains unclear [9]. The median survival time for surgical resection of gastric IM is reported to be 11.0 months, while that for chemotherapy is 4.0 months and that with no treatment is 2.0 months [10]. However, Kato et al. reported no marked difference in outcomes between chemotherapy and chemoradiation for patients with gastric IM [11]. It is generally agreed that gastric IM requires multidisciplinary treatment, including surgery [12].

We encountered a 72-year-old woman with esophageal cancer and a perforated gastric ulcer who underwent palliative resection due to strong invasion of the perforated gastric wall and celiac artery. A biopsy of the gastric ulcerative lesion revealed squamous cell carcinoma, and gastric IM of esophageal cancer was the potential diagnosis. A pathological examination confirmed the cause of gastric perforation to be IM of esophageal cancer. Nivolumab was administered as the indicated secondary therapy for residual tumors. Radiation therapy was also considered, but the residual tumor on CT was just above the celiac artery, and the intestinal tract overlapped in front of the tumor, so the radiologist judged that radiation therapy was difficult because of side effects such as mucositis and hemorrhage. After two cycles of nivolumab, the patient’s tumor showed marked shrinkage on CT, and the patient is still receiving nivolumab 9 months after the surgery. Our findings demonstrate the potential benefits of using ICIs in combination with surgery for esophageal cancer with gastric IM, even in cases where noncurative surgery is performed. The treatment results are comparable to those in previous reports (Table 1). Even in cases such as this one, which require nonhealing factors that may not be completely resected after surgery, surgical treatment may be aggressively considered if the perforation, including the cancer, can be included in the resection area and the patient’s general condition improves after resection of the perforation and reconstruction of the primary tumor, leading to subsequent drug treatment, such as ICI therapy. Currently, the first-line treatment for unresectable advanced and recurrent esophageal cancer is a combination of ICIs and chemotherapy [13, 14]. Even in cases where local invasion is present and considered unresectable, surgery may be considered if local control can be achieved with ICIs. ICIs are expected to become an extremely important treatment strategy for esophageal cancer in the future.

**Table 1:** Postoperative treatment and the prognosis of patients who underwent surgery for gastric IM from esophageal cancer

Author	Year	Age (years)/sex	Operation	Postoperative treatment	Survival time (months)
Yoshizumi et al.	1985	65/M	E+TG	None	ND
Yoshida et al.	1989	64/M	E + PG	Chemotherapy	3
Takano et al.	1989	61/M	E + TG	None	ND
Tajika et al.	1999	67/M	E + LR	None	ND
Uehara et al.	2008	67/M	E + TG	None	11
Nakazawa et al.	2014	59/M	E + PG (+ α)	DCF	8
Okumura et al.	2015	68/M	E + TG	None	14
Hosoda et al.	2018	73/M	TG	None	3

*E* esophagectomy, *TG* total gastrectomy, *PG* proximal gastrectomy, + α left lateral segmentectomy of liver and pancreatosplenectomy, *ND* not described, *IM* intramural metastasis

## Conclusions

Now that ICIs are widely available, long-term survival may be expected with multidisciplinary treatment of surgery plus ICIs, as in the present case with poor prognostic factors. Therefore, more aggressive surgical options may be considered in such cases with noncurative factors.

## Data Availability

The availability of the data used in this study is subject to confirmation by the journal or the authors. For more information on data availability and access procedures, please contact the journal or coresponding author.

## Abbreviations

IM: Intramural metastasis
ICI: Immune checkpoint inhibitor
CT: Computed tomography
